# Kinetics of antimalarial antibodies in children with common haemoglobinopathies in a Tanzanian population

**DOI:** 10.3389/fimmu.2026.1685626

**Published:** 2026-02-18

**Authors:** Alphaxard Manjurano, Nuno Sepulveda, Sixbert Mkumbaye, Taane G. Clark, Arnold Ndaro, Christian William Wang, Louise Turner, Thor G. Theander, Kevin K.A. Tetteh, Eleanor Riley, Franklin W. Mosha, Chris J. Drakeley

**Affiliations:** 1 National Institute for Medical Research, Mwanza Centre, Mwanza, Tanzania; 2 Faculty of Infectious and Tropical Diseases, London School of Hygiene and Tropical Medicine, London, United Kingdom; 3 Joint Malaria Programme, Kilimanjaro Christian Medical Centre, Moshi, Tanzania; 4 Faculty of Mathematics and Information Science, Warsaw University of Technology, Warsaw, Poland; 5 Kilimanjaro Clinical Research Institute, Kilimanjaro Christian Medical Centre, Moshi, Tanzania; 6 Centre for Medical Parasitology, Department of International Health, Immunology & Microbiology, University of Copenhagen and Department of Infectious Diseases, Rigshospitalet, Copenhagen, Denmark; 7 Kilimanjaro Christian Medical University College, Moshi, Tanzania

**Keywords:** antimalarial antibodies, children, haemoglobin (Hb), haemoglobinopathies, Tanzania, malaria

## Abstract

**Introduction:**

Haemoglobin gene mutations have long been associated with protection against malaria, as evidenced by the concordant geographic distribution of these mutations with malaria parasite prevalence and by the reduced risk of severe disease among individuals carrying thalassaemia or sickle cell trait alleles. However, the mechanisms underlying this protection remain poorly understood. Although the precise correlates of immunity to malaria are still debated, antibody-mediated responses are widely considered to play a critical role. In this study, we investigated changes in putatively protective anti-malarial antibody titres in relation to age, malaria infection, and protection in Tanzanian children with and without α^+^-thalassaemia.

**Methods:**

Antibody responses were quantified using a multiplex assay targeting sporozoite antigens (CSP), merozoite antigens (AMA1, MSP1_19_, MSP3, GLURP R0 and R2), and infected red blood cell surface antigens (PfEMP1 groups A, B, and E). A linear mixed-effects modelling framework, assuming a multivariate normal distribution of residuals, was applied to determine whether antibody responses to specific antigens or antigen groups differed by haemoglobinopathy status or were associated with protection from malaria.

**Results:**

In age-adjusted analyses, antibody levels to MSP3 and Group B PfEMP1 exhibited opposing associations with inherited red blood cell disorders: responses were negatively associated with α^+^-thalassaemia and positively associated with sickle cell trait, respectively.

**Conclusion:**

These findings suggest that sickle cell trait may modulate PfEMP1 expression, thereby weakening the adhesion of *Plasmodium falciparum*–infected red blood cells to microvascular endothelial cells, while α⁺-thalassaemia may interfere with the shedding of parasite surface proteins involved in erythrocyte invasion. Collectively, these results provide further insight into the immunological and cellular mechanisms by which haemoglobinopathies confer protection against malaria.

## Introduction

Alpha thalassaemia and other genetically-determined erythrocyte defects were central to the hypothesis proposed by Haldane in the 1940s (1) that the high prevalence of haemoglobinopathies in southern European and African populations resulted from the protection against malaria afforded to carriers of these traits. This hypothesis has subsequently been validated for several haemoglobinopathies, indirectly by concordance between the prevalence of malaria and frequencies of mutant alleles (2, 3), and directly through protection from disease in carriers (2, 4–8).

The distribution of α^+^-thalassaemia and sickle-cell traits has been inversely correlated with altitude (used as a proxy for parasite prevalence) in Melanesia (2) and Tanzania (3). Whilst carriers of α^+^-thalassaemia are protected against severe malaria (9, 10), they receive considerably less—or no—protection against uncomplicated malaria and asymptomatic parasitaemia (11, 12). The mechanism underlying this protection is poorly understood. However, possible hypotheses include the notion that Hb variants allow parasites to display abnormal PfEMP1 on knobs at the surface of *Plasmodium falciparum*-infected red blood cells (PfRBCs), thereby weakening the binding of PfRBCs to microvascular endothelial cells (MVECs) (9). Other theories suggest that haemoglobin (Hb) variants slice the “Gordian knot” of host and parasite interactions to confer malaria protection (reviewed in (13)) or that Hb variants impair cytoadherence, leading to abnormal display of PfEMP1 on the surface of host red blood cells (RBCs) (9, 14). Furthermore, heterozygous β-thalassaemia may enhance the redox stress caused by malaria parasites, inducing its protective effect through the host cell membrane destabilisation (15). It is also possible that affected individuals develop more effective antimalarial immunity (16) or that thalassaemia status affects the intraerythrocytic multiplication of *P. falciparum* (17).

Whilst the exact mechanism of immunity to malaria is debated, it is generally accepted that antibodies play a key role. Both the breadth and the magnitude of the antibody response (18, 19), as well as the sequential development of antibodies to variant antigens expressed on the surface of infected red blood cells (iRBC) (20), are believed to be important. Moreover, a study in Mali found that HbS and HbC heterozygosity neither increases the magnitude nor the breadth of antibody responses to a diverse range of *P. falciparum* antigens that were studied (21, 22).

Although two studies found no difference in levels of antibodies to single malaria antigens between individuals with and without thalassaemia (18, 23), a recent study in Sri Lanka found higher antibody responses to both *P. falciparum* and *Plasmodium vivax* in individuals with β-thalassaemia than in nonthalassaemic controls (16). Moreover, in a large immunogenetic study in Northeast Tanzania, we observed significantly higher prevalences and titres of antibodies to various malaria blood-stage antigens in α-thalassaemics than in nonthalassaemics (manuscript in preparation). The biological mechanisms underlying the apparently increased immunogenicity of malaria antigens in thalassaemic individuals are unknown, but three nonexclusive hypotheses exist: (1) in α-thalassaemics, the mechanical destruction of iRBCs that leads to anaemia is slowed or prevented (24), resulting in carriage of low levels of parasites and subsequent protective premunition; (2) as observed for sickle-cell trait (25), altered expression of malaria proteins on the surface of thalassaemic iRBCs reduces their ability to sequester in peripheral tissues, leading to more rapid clearance in the spleen (26), where they induce more effective immune responses that clear iRBCs from the blood (13, 27); or (3) haemoglobin variants interfere with the export of *P. falciparum* erythrocyte membrane protein 1 (PfEMP1) to the surface of the host red blood cell, thereby enhancing the adaptive immune response to clear iRBCs (13).

The hypothesis is that haemoglobinopathies accelerate the acquisition of humoral immunity to malaria by profiling Immunoglobulin G (IgG) responses of Tanzanian children to a number of *P. falciparum* proteins expressed during pre- and erythrocytic stages of parasite development. This study aimed to investigate the relationship between individuals carrying alleles for thalassaemia and sickle-cell traits and malarial antibody responses. In Northeast Tanzania, we have described epidemiological, clinical, and parasitological characteristics of malaria across a range of transmission settings, where altitude acts as a proxy for malaria transmission intensity (28–30).

## Methods

### Study area and subjects

**Figure 1:**
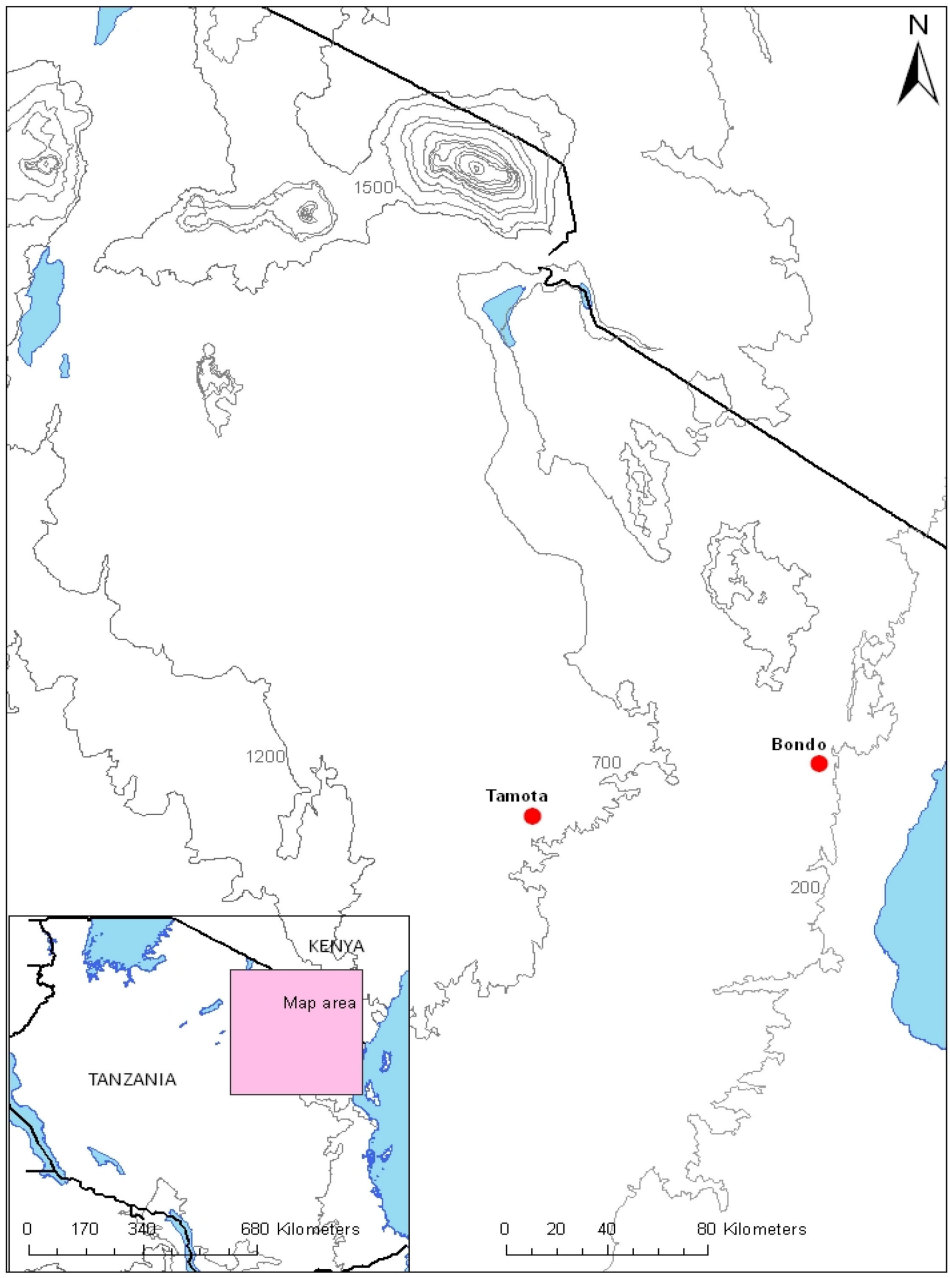
Map of the study villages. The villages involved in this study are indicated by red dots.

The study was carried out in the villages of Bondo (Handeni district, altitude: 300 m, 5.55°30′0″ S; 38°0′0″ E, malaria prevalence: 45%, a high malaria transmission village (24), α^+^-thalassaemia prevalence: ~ 40%) and Tamota (Lushoto district, altitude 1,500 m, malaria prevalence: 6%, α^+^-thalassaemia prevalence: 23%, a low malaria transmission village) from May 2011 to January 2012 (**Figure 1**).

The Bondo area has a bimodal rainfall pattern, with a long rainy season lasting from March to May, corresponding to the high malaria transmission season, and short rains between October and December. The dry season lasts from June to September. The Tamota area also exhibits a bimodal pattern of mean monthly rainfall, with maximum rainfall occurring in April and May, and again in November/December.

The main malaria vectors are *Anopheles gambiae s.s.*, *A. arabiensis*, and *A. funestus*. About 95% of the population in the Tamota area belongs to the Sambaa ethnic group, whereas one-third of individuals in Bondo belong to the Zigua (Tanzanian Census, 2012). The main economic activities include farming (oranges, maize, banana plantations, beans, and cassava), livestock keeping, and beekeeping.

### Recruitment of study participants

Children aged 2–9 years were recruited from those presenting to a health central facility over a period of 2 to 3 weeks. Once consented, they were sampled on a first-come, first-served basis. Demographic, anthropometric, and clinical data were recorded, and a 0.5-ml finger-prick blood sample was collected. Minor ailments were treated by clinical staff, whilst individuals with more serious health problems were referred to an appropriate health facility.

### Follow-up and case detection

**Figure 2:**
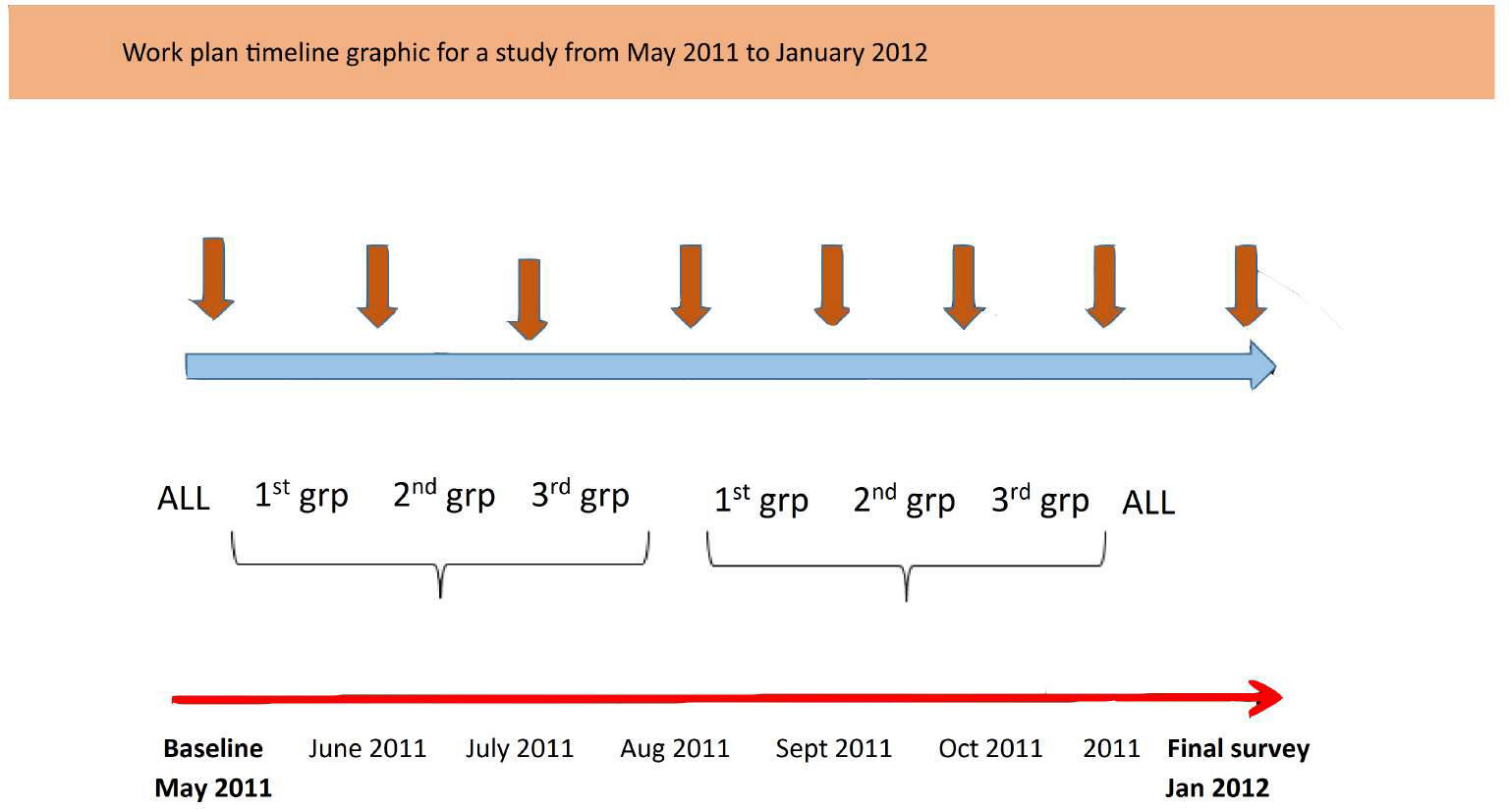
Schematic diagram showing the sampling procedure.

Following recruitment, study participants were assigned to three groups for follow-up: one group was sampled 1 and 4 months after enrolment, the second group was sampled 2 and 5 months after enrolment, and the third group was sampled 3 and 6 months after enrolment. All participants were sampled during the final survey in month 7 (**Figure 2**). During an unscheduled visit, parents were requested to bring their children to the clinic if the child developed a fever or became unwell; otherwise, the children were expected to attend the health facility on a scheduled visit for clinical examination and sample collection. A clinical officer was on 24-h duty and collected medical information on standardised forms. Axillary temperature was measured using an electronic thermometer, and malaria rapid diagnostic tests (RDT) (Paracheck Pf^®^, Orchid Biomedical Systems, Mumbai, India) were administered for children with guardian-reported fever during unscheduled and scheduled visits. Blood smears were prepared from samples collected from all study participants during scheduled visits. Those with positive RDT results were treated in accordance with national guidelines; uncomplicated malaria was treated with artemether-lumefantrine (Novartis Pharma, Basel, Switzerland). This study employed RDT during unscheduled visits because of their ease of use in the study areas compared with microscopy, which required electricity. Hb levels were measured only during the final survey. Children with Hb levels of < 11 g/dl received iron supplementation, whereas those with severe anaemia (< 5 g/dl) were referred to the nearest health facility.

### Laboratory methods

Approximately 0.5 ml of finger-prick blood was collected from participants into EDTA vacutainers. A blood film was prepared, and RDT and haemoglobin levels were measured using a haemocue device. Giemsa-stained blood films were examined by oil-immersion microscopy. One hundred fields were screened before a blood smear was deemed to be negative; if parasites were observed, they were counted against 200 white blood cells (WBCs). All slides were read by two independent microscopists; if results were discordant, a third reading was performed by a different microscopist. The majority result was accepted for slide positivity, and the mean of all three readings was used to estimate parasite density. Samples were spun at 1,479 rcf for 10 min, and the plasma was removed and stored for future analysis of antibodies. DNA was extracted and purified from the blood cell pellet using a Nucleon kit, according to the manufacturer’s instructions (http://www.tepnel.com).

### Ethics statement

All DNA samples were collected and genotyped following signed and informed consent from a parent/guardian. Ethics approval for all procedures was obtained from both Kilimanjaro Christian Medical Centre Research Review ethics (No. 380), the London School of Hygiene and Tropical Medicine (LSHTM) (No. 2087), and the Tanzanian National Institute for Medical Research (NIMR/HQ/R.8a/Vol.IX/392).

### Red blood cell changes and G6PD deficiency polymerase chain reaction genotyping

The α^3.7^-thalassaemia deletion was typed by polymerase chain reaction (PCR) as previously described (31). Sickle-cell variants and Glucose-6-phosphate dehydrogenase (G6PD) deficiency were genotyped using PCR followed by restriction fragment length polymorphisms (RFLP), as described elsewhere by Modiano and Fanello, respectively (32, 33).

### PCR for subpatent parasite carriage

A nested PCR was used to amplify species-specific sequences of the small subunit ribosomal ribonucleic acid (18S SSU rRNA) genes of *P. falciparum*, as previously described (34, 35). DNA from a culture of *P. falciparum* (3D7 strain) served as a positive control, whilst DNA from the blood of an individual never exposed to malaria and no-template controls were included in each set of PCRs for quality control.

### Luminex assay

Antibodies to a panel of sporozoite, merozoite, and infected red blood cell surface antigens were analysed using a bead-based assay on the BioPlex^100^ (BioRad, Hercules, CA, USA) system, as described elsewhere (36). The panel included 10 recombinant PfEMP1 proteins (**Supplementary Table S1**), the C-terminal region of MSP3, and the R0 and R2 of glutamate-rich protein (GLURP), as previously described (37, 38), together with AMA1, Merozoite surface protein 1 (MSP1), and CSP antigens (39, 40). The reader was set to detect a minimum of 100 beads with an identical unique detection signal, and the results were expressed as median fluorescent intensity (MFI). To determine whether the human IgG detection antibody bound nonspecifically to the coated beads, the beads were analysed against naïve plasma samples from Danes and British individuals who had never been exposed to malaria, as previously optimised by Turner et al. (41).

### Statistical analysis

To test putative haemoglobinopathy genotypic differences between the Tamota and Bondo populations, we used Pearson’s Chi-square (*χ*^2^) test for two-dimensional contingency tables. The association of HbS, G6PD, and α-thalassaemia with RDT and PCR outcomes was assessed using logistic regression for binary variables. Specifically, we considered a “null” model that included the main effects of gender, age (in years), village (Tamota versus Bondo), ethnicity (Wabondei, Wasambaa, Wazigua, and others), and net usage (never, sometimes, and always). We then built seven genetic models on top of this: three models encompassing the main effects of α-thalassaemia, G6PD, and HbS separately; three models considering the main effects of two of these polymorphisms; and a final model including the main effects of all these polymorphisms. These genetic models were compared to the null using the likelihood ratio test. The corresponding −log_10_(*p*-value) was reported as a measure of genetic association. Higher values of −log_10_(*p*-value) indicate stronger statistical evidence for a given genetic model compared with the null model. The association of these three polymorphisms with different *P. falciparum*-specific IgG antibodies was examined using longitudinal data. For statistical convenience, antibody data were log_10_-transformed to approximate normal distributions. Transformed data were then analysed using a linear mixed-model approach, assuming a multivariate normal distribution for the residuals. For each antibody dataset, we fitted a null longitudinal regression model including the fixed effects of gender, age, village, survey, and ethnicity, and a Gaussian random effect for each individual to account for the correlation between measurements from the same person. We then compared this null model to seven genetic models, including all possible combinations of effects of α-thalassaemia, G6PD, and HbS on the outcome, as done for PCR positivity data. The best genetic model was defined as the one providing the minimum *p*-value of the likelihood ratio test when compared with the null models. A genetic model was considered significant if the corresponding *p*-value of the likelihood ratio test was < 0.05 (or, alternatively, −log_10_[*p*-value] > 1.30). All analyses were performed using the R statistical software (http://www.r-project.org). In particular, we used the *lme4* package to perform the longitudinal data analysis.

## Results

### Baseline characteristics of the study participants

During 4 weeks in May and June 2011, a total of 767 and 868 individuals in Bondo and Tamota, respectively, were enrolled. Study participants were aged 2 to 9 years. Follow-up at scheduled cross-sectional visits was conducted for children throughout the 8-month study. **Table 1** shows baseline characteristics.

**Table 1:** Baseline and clinical characteristics.

Variables	Bondo (*N* = 767)		Tamota (*N* = 868)	
*Age^a^ (mean, SD)*	(5.5)	(2.3)	(5.3)	(2.1)
*Gender (girls)*	385	50.3%	442	51.0%
*Ethnicity*	*n*	%	*n*	%
Wazigua	423	55.7	30	3.5
Wasambaa	87	11.5	795	91.6
Wabondei	17	2.2	2	0.2
Wabena	66	8.7	–	–
Wapare	27	3.5	17	2.0
Other	140	18.4	24	2.7
Malaria prevalence at enrolment				
mRDT (%, 95% CI)		23.3 (20.5–26.4)		0.8 (0.3–1.7)
Microscopy (%, 95% CI)		18.8 (16.2–21.7)		0.2 (0.03–0.8)
Subpatent parasites^b^ (*n*, %)	261	46.4	18	2.2
Bednet use last night^c^ (*n*, %)	445	58.8	452	52.1
Hb (g/dl; mean ± SD)^d^		11.4 (± 1.4)		12.0 (± 1.3)

a In years.

b Parasite by PCR after excluding patent parasites.

c Some participants did not respond.

d Haemoglobin level at the end of the surveys.

### Prevalence of RBC polymorphisms and their impact on RDT and PCR outcomes

With respect to the α-thalassaemia locus, the genotypic distribution of the Bondo population was estimated at 46.6%, 39.1%, and 14.3% for wild-type, −α/αα, and −α/−α genotypes, respectively (**Table 2**). In Tamota, we observed a statistically significant increase of wild-type genotypes (54.5%) along with a decrease in −α/−α genotypes (8.6%) (*p* = 0.0002). For the sickle-cell polymorphism, the Bondo population had higher levels of both the sickle cell trait (HbAS) and sickle cell disease (HbSS) genotypes (2.9% and 9.5%, respectively) compared with Tamota (2.1% and 2.0%, respectively) (*p* < 0.001). Finally, the prevalence of *G6PD*A− in girls (hetero- and homozygotes) was 14.7% and 13.4% in Bondo and Tamota, respectively. In boys (hemizygotes), the prevalence was 10.6% and 9.0% for the Bondo and Tamota populations, respectively.

**Table 2:** Red blood cell polymorphisms.

Variables	Bondo^a^		Tamota^a^		*P*-*value*
*RBCs changes*	*N*	% (95% CI)	*N*	% (95% CI)	
α-Thalassaemia					
Normal	339	46.6 (43.0–50.2)	464	54.4 (51.1–57.7)	0.0002
Heterozygous	284	39.1 (35.6–42.7)	316	37.1 (33.8–40.4)	
Homozygous	104	14.3 (11.8–16.9	73	8.5 (6.7–10.4)	
Haemoglobin type					
AA	659	87.6 (85.2–89.9)	788	95.9 (94.6–97.3)	< 0.001
AS	22	2.9 (1.7–4.2)	17	2.1 (1.1–3.1)	
SS	71	9.5 (7.4–11.6)	16	2.0 (1.0–3.0)	
G6PDA−					
Girls					
Normal	324	85.3 (81.7–88.8)	362	86.6 (83.3–89.9)	0.770
Heterozygous	44	11.6 (8.4–14.8)	46	11.0 (8.0–14.0)	
Homozygous	12	3.1 (1.4–4.9)	10	2.4 (0.9–3.9)	
Boys					
Normal	328	89.4 (86.3–92.5)	363	91.0 (88.2–93.8)	0.532
Hemizygous	39	10.6 (7.5–13.7)	36	9.0 (6.2–11.8)	

a Total number for each of the genotyped RBC variants.

**Figure 3:**
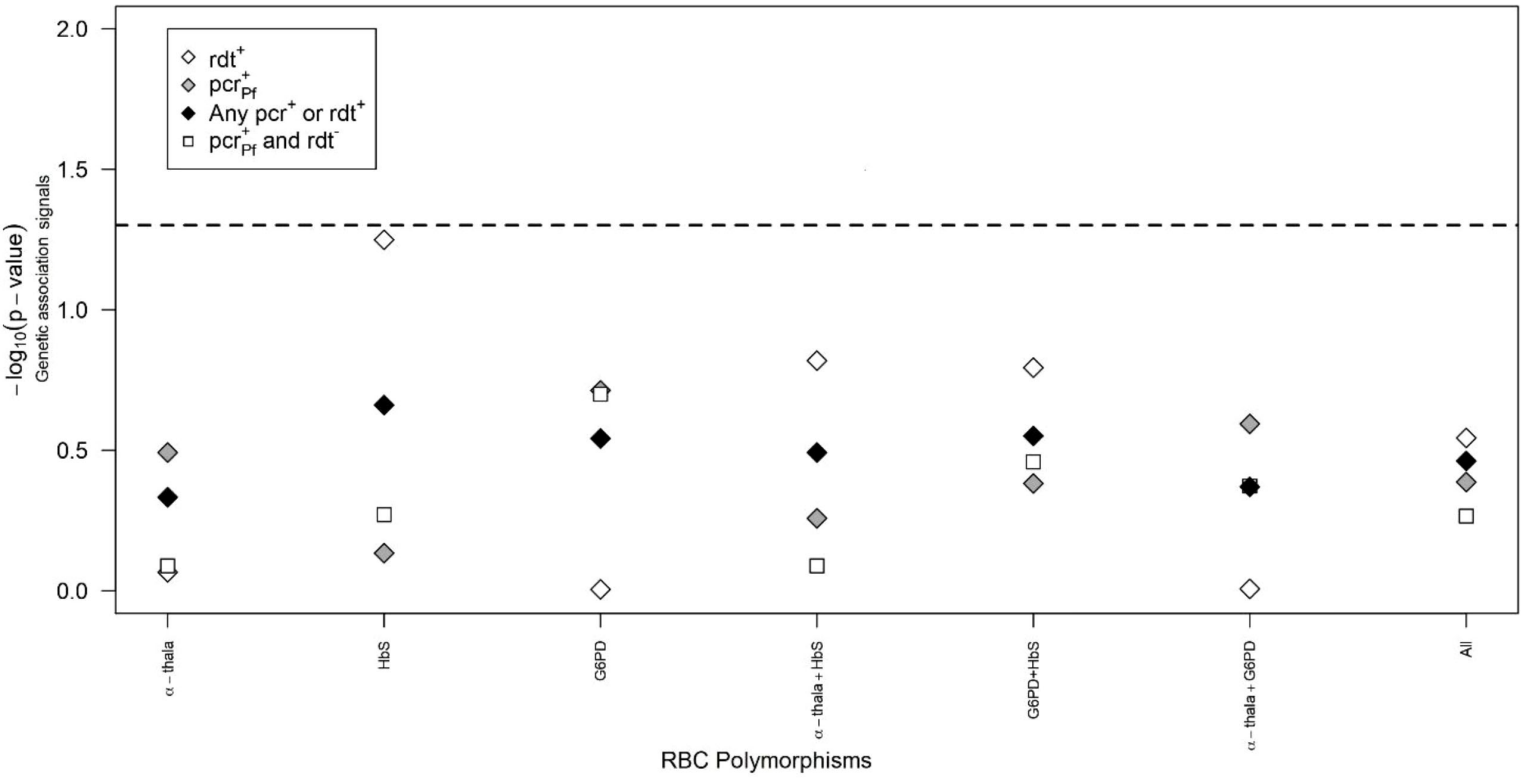
Analysis of genetic association with PCR and RDT positivity outcomes. Impact of HbS, α-thalassaemia, and G6PD polymorphisms on different parasite-specific IgG antibodies. In this analysis, models with genetic effects of these polymorphisms are considered better than those without such genetic effects when the corresponding association signals are above the horizontal dashed lines, which refer to −log_10_(0.05) associated with the 5% significance level. The *x*-axis refers to all tested models against the data.

We performed an association analysis of RBC polymorphisms on RDT and PCR positivity outcomes (RDT positivity, PCR positivity, any PCR or RDT positivity, and any PCR positivity and RDT negativity). Before adjustment for confounders, several strong association signals were observed between the RBC polymorphisms and the different parasite positivity phenotypes (**Supplementary Figure S1**). However, after adjusting for gender, village, age, ethnicity, and net use, these genetic association signals became nonsignificant (**Figure 3**). Following adjustment, the strongest association signal was obtained for HbS on RDT positivity (−log_10_(*p*-value) = 1.22), but it did not align with other parasite positivity phenotypes, as the corresponding association signals decreased to 0.24, 0.66, and 0.27 for PCR positivity, any RDT or PCR positivity, and any RDT and PCR positivity, respectively. We conclude that RBC polymorphisms may not be associated with parasite infection in the study area.

### HbS polymorphisms and α-thalassaemia loci are moderately associated with the *P. falciparum*-specific IgG responses

**Figure 4:**
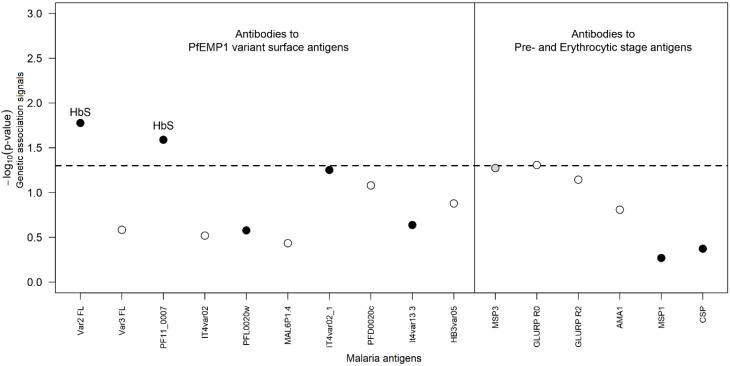
Genetic association analysis of IgG antibody to different parasite proteins. The *x*-axis refers to the different tested antibodies, and each dot represents the best genetic model (with covariates and putative genetic effects of RBC polymorphisms) for the corresponding data (black-filled circles, HbS; grey-filled circles, α-thalassaemia; empty circles, G6PD).

To assess the association of sickle-cell gene, *G6PD*, and α-thalassaemia with humoral immune responses, we performed a longitudinal data analysis on a random subset of 150 individuals (50 from Tamota and 100 from Bondo) to avoid familial clustering. Distinct IgG antibodies were measured at eight different time points, four per individual. In this dataset, the prevalence of different RBC polymorphisms was 35.5% (53/150), 13.9% (20/144 plus six missing genotypes), and 14.8% (22/149 plus one missing genotype) for α^+^-thalassaemia, HbS^+^, and G6PD^+^, respectively. Adjusting for confounders, the longitudinal analysis accounted for the correlation between measurements from the same individual by including random effects in the corresponding statistical models. In this analysis, we found statistical evidence for moderate effects of HbS on the kinetics of IgG to the PfEMP1 antigens IT4var04 (var2 FL) and PF11_0007 (−log_10_[*p*-value] = 1.78 and 1.50, respectively; **Figure 4**). For both antigens, individuals with HbAS and HbSS genotypes tended to show an increase of 0.208 and 0.204 orders of magnitude in circulating antibodies to IT4var04 (var2 FL) and PF11_0007, respectively, compared with HbAA (**Table 3**). Two borderline associations were also observed between α-thalassaemia and IgG antibodies to the MSP3 antigen (−log_10_[*p*-value] = 1.22) and between G6PD and the GLURP R0 antigen (−log_10_[*p*-value] = 1.31; **Supplementary Table S2**). Although not reaching statistical significance, these RBC polymorphisms were associated with a decrease in the number of circulating antibodies to the respective antigen.

**Table 3:** Effect of sickle-cell trait on antibody response to PfEMP1 antigens.

Parameters	var2_FL		PF11_0007	
	Estimate (SE)	*P*-value	Estimate (SE)	*P*-value
Intercept	2.526 (0.115)	< 0.001	2.140 (0.120)	< 0.001
Survey				
Second	0.125 (0.050)	0.012	0.129 (0.047)	0.006
Third	0.126 (0.064)	0.049	0.132 (0.059)	0.025
Fourth	0.197 (0.066)	0.003	0.183 (0.055)	0.001
Fifth	0.141 (0.047)	0.003	0.116 (0.046)	0.012
Sixth	0.190 (0.050)	< 0.001	0.203 (0.051)	< 0.001
Seventh	0.088 (0.057)	0.122	0.088 (0.056)	0.117
Eight	0.087 (0.035)	0.013	0.088 (0.034)	0.009
Village				
Tamota	0.085 (0.084)	0.310	− 0.012 (0.087)	0.886
Gender				
Boys	0.103 (0.059)	0.081	0.097 (0.062)	0.118
Age (years)	0.074 (0.014)	< 0.001	0.098 (0.014)	< 0.001
Ethnicity				
Wabondei	− 0.141 (0.191)	0.462	− 0.268 (0.202)	0.185
Wazigua	0.170 (0.086)	0.049	0.162 (0.091)	0.075
Other	− 0.048 (0.093)	0.602	− 0.016 (0.098)	0.867
HbAS + HbSS effect	0.208 (0.086)	0.016	0.204 (0.091)	0.025

Estimation of the longitudinal models for the HbS effect on IgG antibodies to var2 FL and PF11_007 antigens, where the HbS effects for heterozygotes and homozygotes are considered together.

*Var2 FL*, Var2 full length; *SE*, standard error; *HbAS*, heterozygous sickle-cell anaemia; *HbSS*, homozygous sickle-cell anaemia.

## Discussion

This study tested the hypothesis that haemoglobinopathies accelerate the acquisition of humoral immunity to malaria by profiling IgG responses of Tanzanian children to several *P. falciparum* proteins expressed during pre- and erythrocytic stages of parasite development.

Antibodies against variant surface antigens, PfEMP1 and RIFIN, as well as MSP3 and GLURP, are acquired after only one infection (41). Here, we assessed the presence of antibodies to a small panel of different PfEMP1, including group B/A PfEMP1 containing domain cassette 8 (DC8), which, together with group A PfEMP1, is associated with severe malaria in children (42), and antibodies are acquired first to these groups (20). Furthermore, a study by Lavstsen et al. (43) indicated that parasites causing severe malaria express a subset of PfEMP1, showing differential expression of PfEMP1 genes, with group A being the least expressed. In this study, we found a significant association with the recognition of a great magnitude of antibodies to a group B PfEMP1 in children with sickle-cell trait compared to normal individuals. This observation may imply that the host Hb types are involved in modulating PfEMP1 expression. A study carried out by Cham et al. (20) suggested that individuals with limited exposure to malaria initially respond to parasites expressing PfEMP1 groups A and B/A, allowing parasites expressing less pathogenic PfEMP1 to dominate during later infections. The findings of the current study and previous studies by Cham et al. (20) and Lavstsen et al. (43) imply different sequential acquisition of antibodies to PfEMP1, which may reflect differences in the structure of the var gene and, consequently, differences in how they are transported to the surface of the RBC (20).

Furthermore, our findings are consistent with those of Verra et al. (44), who reported that *P. falciparum*-specific IgG responses are enhanced in HbAS and Haemoglobin C (HbAC) trait children in Burkina Faso. Our findings on sickle-cell status, however, differ from observations in a study conducted in Mali by Tan et al. (21), which reported that HbAS and HbAC did not independently enhance *P. falciparum*-specific antibody responses to several tested *P. falciparum* proteins expressed during exoerythrocytic and erythrocytic stages of the life cycle. This discrepancy can be explained by the fact that they did not use correctly folded antigens, as discussed elsewhere (45).

The increased antibody levels to VAR2 Chondroitin Sulfate A (VAR2CSA) in sickle-cell trait carrier individuals may be explained by the fact that most variants are expressed by the parasite population at the onset of the blood-stage infection to increase the survival in a new host (43, 46, 47). We also cannot rule out the possibility of cross-reactive antibodies induced by the other PfEMP1 variants (20, 48, 49). It should be noted that VAR2CSA has been a critical mediator in the pathogenesis of placental malaria (50), and VAR2CSA-specific antibodies (IgGs) are important for protecting pregnant women (49, 51, 52).

In age-adjusted analyses, we found that antibody levels to MSP3 differed between α-thalassaemic and nonthalassaemic individuals through the study period, which encompassed times of high and low transmission. Alpha^+^ thalassaemia may interfere with the shedding of peripheral surface proteins (13), which could explain the negative correlation between MSP3 antibodies and being thalassaemic. *P. falciparum* peripheral surface proteins, such as MSP3, MSP1, MSP7, Serine repeat antigens (SERA4), and SERA5, have been suggested to play a role in initial invasion events (53).

A study by Turner et al. (41) showed that antibodies to polymorphic antigens expressed during parasite erythrocytic stages are essential mediators of immunity and are acquired early in life; submicroscopic parasites also contribute to high antibody levels in individuals (54). The negative correlation between G6PD-deficient individuals and antibodies to GLURP is consistent with observations from a study in Senegal by Courtin et al. (55), which found that G6PD A− carriers had a smaller increase in IgG3 levels to MSP2/FC27 and MSP2/3D7 than noncarriers. This effect can be explained by reduced exposure to malarial antigens related to this genetic variant, leading to weaker stimulation of specific antibody responses, as previously reported (55).

This study has several strengths that allow a more comprehensive assessment of the effect of haemoglobinopathies on the acquisition of *P. falciparum*-specific humoral immunity. Importantly, we studied IgG responses to a diverse set of *P. falciparum* proteins to enable analyses of subsets of *P. falciparum*-specific IgG responses by life-cycle stage and subcellular location within iRBCs. In addition, the study was designed to minimise differences in *P. falciparum* exposure between children who differed by α-thalassemia status, taking into account altitude, a proxy for malaria transmission in the study area (28).

This study has some limitations: (i) we measured IgG responses to a limited set of malaria antigens, and other proteins may be relevant to malaria protection among children with haemoglobinopathies (56, 57); (ii) the Hb effect may be more pronounced in the youngest children than in the age group considered in this study; (iii) we could not employ microscopy for diagnosing malaria during the unscheduled visits, which could have provided a more robust measure of infection status and parasite burden, potentially enriching the correlation analysis with antibody levels.

In summary, our data suggest that haemoglobinopathies (being α-thalassaemics or HbAS/HbSS) significantly modulate IgG responses specific to *P. falciparum* proteins. The findings of this study may provide new insight into the mechanisms by which haemoglobinopathies confer protection against malaria. Further studies with larger sample sizes and in different epidemiological settings are needed to replicate these findings and better understand the relationships among α-thalassaemics, HbAS/HbSS, and acquired immunity to malaria. We also propose additional studies using functional assays to validate hypotheses generated by the findings of this study.

## Data Availability

The raw data supporting the conclusions of this article will be made available by the authors, without undue reservation.

## Supplementary material

Supplementary Figure 1 Analysis of genetic association with PCR and RDT positivity outcomes.

Supplementary Table 1 The 10 recombinant DBL domains used for the study.

Supplementary Table 2 Effects of RBC polymorphisms on the IgG antibodies to MSP3 antigen.
