# Supplementary figures and images for: Kinetics of antimalarial antibodies in children with common haemoglobinopathies in a Tanzanian population
Source: Front Immunol. 2026 Feb 18;17:1685626. doi: 10.3389/fimmu.2026.1685626 (PMC12957101; doi:10.3389/fimmu.2026.1685626)

**Supplementary Figure S1: Analysis of Genetic Association with PCR and RDT positivity outcomes**


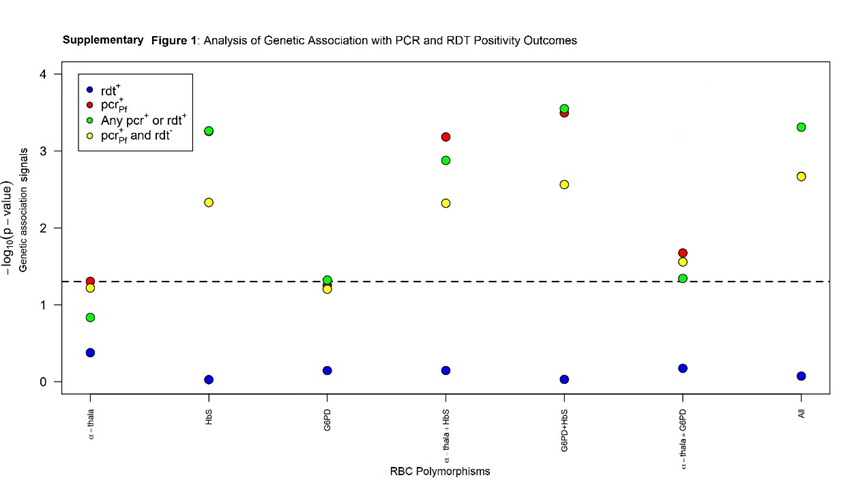

Supplement: Supplementary Figure 1 — Analysis of genetic association with PCR and RDT positivity outcomes. [file DataSheet1.docx]
