# Supplementary material for: Kinetics of antimalarial antibodies in children with common haemoglobinopathies in a Tanzanian population
Source: Front Immunol. 2026 Feb 18;17:1685626. doi: 10.3389/fimmu.2026.1685626 (PMC12957101; doi:10.3389/fimmu.2026.1685626)
Supplement: Supplementary Table 1 — The 10 recombinant DBL domains used for the study. [file Table1.docx]

**Supplementary Table S1**: **The 10 recombinant DBL domains used for the study**

| *Pf*EMP1 | *Pf*EMP1  Group*^a^* | *Pf*EMP1 Annotation^b^ | | | | | | |
| --- | --- | --- | --- | --- | --- | --- | --- | --- |
| IT4var04 (Var 2 FL) | E | DBL | DBL | CIDR | DBL | DBLε | DBLε | DBLε |
| PFI1820w (Var 3 FL) | A | DBLα | DBLε |  |  |  |  |  |
| PF11_0007 | B | DBLα | CIDR | DBLδ | CIDR | ATS |  |  |
| IT4var02 | A | DBLγ | DBLδ | CIDR | DBLβ |  |  |  |
| PFL0020w | B | DBLγ | DBLξ | DBLε |  |  |  |  |
| MAL6P1.4 | B | DBLε | DBLε | DBLε |  |  |  |  |
| IT4var02 | A | DBLδ | CIDR |  |  |  |  |  |
| PFD0020c | A | DBLβ | DBLγ |  |  |  |  |  |
| It4var13 | B | DBLα | CIDR | DBLβ | DBLδ | CIDR | DBLγ | DBLξ |
| HB3var05 | A | DBLα | CIDR |  |  |  |  |  |

*a* Grouping of native PfEMP1 according to Lavstsven et al. [[38](#_ENREF_38)].

*b* Annotation of the domains (position and type) in the native molecule.

FL refers to Full Length
