# Supplementary material for: Kinetics of antimalarial antibodies in children with common haemoglobinopathies in a Tanzanian population
Source: Front Immunol. 2026 Feb 18;17:1685626. doi: 10.3389/fimmu.2026.1685626 (PMC12957101; doi:10.3389/fimmu.2026.1685626)
Supplement: Supplementary Table 2 — Effects of RBC polymorphisms on the IgG antibodies to MSP3 antigen. [file Table2.docx]

**Supplementary Table S2: Effects of RBC polymorphisms on the IgG antibodies to MSP3 antigen**

Estimation of the longitudinal models for the α^+^-thalassaemia effect on the IgG antibodies to MSP3 antigen, and the G6PD effect on the IgG antibodies to GLURP R0 antigen, where the genetic effects are for heterozygotes and homozygotes taken together.

| Parameters | | α-thalassaemia and MSP3 | |  | G6PD and GLURP R0 | | |
| --- | --- | --- | --- | --- | --- | --- | --- |
|  |  | Estimate (SE) | p-value |  | Estimate (SE) | p-value |  |
| Intercept | | 2.096 (0.186) | <0.001 |  | 2.984 (0.156) | <0.001 |  |
| Survey | |  |  |  |  |  |  |
|  | Second | 0.233 (0.073) | 0.001 |  | 0.186 (0.057) | 0.001 |  |
|  | Third | 0.458 (0.094) | <0.001 |  | 0.211 (0.071) | 0.003 |  |
|  | Fourth | 0.419 (0.086) | <0.001 |  | 0.015 (0.068) | 0.820 |  |
|  | Fifth | 0.198 (0.073) | 0.006 |  | 0.118 (0.056) | 0.037 |  |
|  | Sixth | 0.223 (0.080) | 0.005 |  | -0.003 (0.062) | 0.968 |  |
|  | Seventh | 0.089 (0.087) | 0.312 |  | -0.045 (0.068) | 0.510 |  |
|  | Eight | 0.075 (0.053) | 0.159 |  | 0.006 (0.041) | 0.875 |  |
| Village | |  |  |  |  |  |  |
|  | Tamota | -0.680 (0.136) | <0.001 |  | -0.337 (0.112) | 0.003 |  |
| Gender | |  |  |  |  |  |  |
|  | Males | 0.021 (0.099) | 0.829 |  | -0.234 (0.081) | 0.004 |  |
| Age, in years | | 0.059 (0.022) | 0.007 |  | 0.032 (0.019) | 0.085 |  |
| Ethnicity | |  |  |  |  |  |  |
|  | Wabondei | -0.069 (0.142) | 0.626 |  | 0.163 (0.119) | 0.170 |  |
|  | Wazigua | -0.672 (0.318) | 0.034 |  | -0.897 (0.261) | 0.001 |  |
|  | Other | -0.187 (0.153) | 0.220 |  | 0.049 (0.126) | 0.697 |  |
| Genetic effect | | -0.196 (0.104) | 0.059 |  | -0.236 (0.119) | 0.048 |  |
